# Oral squamous cell carcinoma proliferative phenotype is modulated by proanthocyanidins: a potential prevention and treatment alternative for oral cancer

**DOI:** 10.1186/1472-6882-7-22

**Published:** 2007-06-19

**Authors:** Michael King, Kourt Chatelain, Dustin Farris, Dayne Jensen, Jason Pickup, Aaron Swapp, Susan O'Malley, Karl Kingsley

**Affiliations:** 1 Department of Biomedical Sciences, School of Dental Medicine, University of Nevada, Las Vegas, USA

## Abstract

**Background:**

Despite the recently reported drop in the overall death rate from cancer, the estimated survival rate and number of deaths from oral cancer remain virtually unchanged. Early detection efforts, in combination with strategies for prevention and risk-reduction, have the potential to dramatically improve clinical outcomes. The identification of non-toxic, effective treatments, including complementary and alternative therapies, is critical if the survival rate is to be improved. Epidemiologic studies have suggested a protective effect from certain plant-derived foods and extracts; however, it has been difficult to isolate and identify the compounds most responsible for these observations. The primary purpose of this study was to investigate the response of human oral squamous cell carcinoma (OSCC) to proanthocyanidin (PAC), a plant-derived compound that may inhibit the progression of several other cancers.

**Methods:**

Using a series of *in vitro *assays, we sought to quantify the effects of PAC on OSCC, cervical carcinoma, and non-cancerous cell lines, specifically the effects of PAC on cell proliferation. Recent data suggest that infection with the human papillomavirus (HPV) may also modulate the proliferative potential of OSCC; therefore, we also measured the effects of PAC administration on HPV-transfected OSCC proliferation.

**Results:**

Our results demonstrated that PAC administration was sufficient to significantly suppress cellular proliferation of OSCC in a dose-dependent manner. In addition, the increased proliferation of OSCC after transfection with HPV 16 was reduced by the administration of PAC, as was the proliferation of the cervical cancer and non-cancerous cell lines tested. Our results also provide preliminary evidence that PAC administration may induce apoptosis in cervical and oral cancer cell lines, while acting merely to suppress proliferation of the normal cell line control.

**Conclusion:**

These results signify that PAC may be a compelling candidate for testing in both animal and human models. Furthermore, these data provide adequate justification for elucidating the divergent mechanisms of PAC-induced proliferation, inhibition, and apoptosis among these and other cell lines.

## Background

More than 30,000 people are diagnosed with oral cancer annually in the United States, and due to a poor survival rate that has remained relatively static, of those who are diagnosed, approximately 7,500 die of this disease each year [1]. Many of these patients will subsequently develop regional tumors, as a result of spreading of the disease to other organs and lymph nodes [2].

Risk factors are anything that significantly increases the chance of a person developing a disease, such as oral cancer. Scientists now agree that the use of tobacco and alcohol, human papillomavirus (HPV) infection, and in the case of lip cancers, exposure to ultraviolet light, are risk factors that increase the likelihood of developing oral cancers [3]. Thus, many oral cancers are believed to have a multifactorial etiology in which environmental and genetic cofactors influence the development and progression of the disease. Recent evidence suggests that among these factors, nutrition may play an important role in retarding the development and progression of oral cancers [4,5]. A recent meta-analysis examining the association between fruit and vegetable consumption and the incidence of oral cancer, found a nearly 50% reduction in oral cancer risk for each additional portion of fruit or vegetable consumed per day [6].

It is generally accepted that fruit and vegetable intake may be correlated with a reduction in the incidence of oral cancers even among tobacco and alcohol users. The knowledge gained thus far, however, does not provide enough specific detail upon which to base effective or comprehensive nutrition or dietary intervention recommendations. More detailed information is needed to identify the active components of fruits and vegetables, to evaluate dose-response relationships, and to adjust for potential confounders. The stratification of these studies, for example, has revealed that citrus fruit consumption was associated with a larger protective effect against oral cancer than overall fruit consumption, and was greater than green- or overall vegetable consumption [6].

Based upon these and other observations, a growing interest has been developing in alternative and complementary medicine to identify botanical and dietary components for their chemopreventive and chemotherapeutic potential. One such class of compounds, the proanthocyanidins (PACs), are polyphenolic compounds that are highly concentrated in certain dietary fruits and nuts, such as grapes, blueberries, cranberries, and almonds, as well as cacao beans and chocolate [7-10].

PACs belong to a larger class of abundant, plant-derived compounds, flavonoids, which appear to provide many beneficial health effects, largely due to their antioxidant properties. PACs have been shown to protect against oxidative stress and tobacco-induced DNA damage, and to exhibit selective cytotoxicity against some human cancers, including breast, lung, prostate, and gastric carcinomas [11-15]. Extracts from grape peels, grape seeds, and black raspberries, sources that contain high concentrations of PAC, have demonstrated selective suppression of tumorigenic phenotypes in oral cancers, specifically in oral squamous cell carcinomas (OSCC) [16,17].

The possible mechanisms of PACs effects on tumor proliferation are complex and are likely to involve multiple pathways, which may differ according to the type of cancer. For example, PAC derived from grape seed, green tea, and lowbush blueberry, exhibited broad, anti-proliferative properties against multiple cell lines, including colon [18], prostate [14,19-21], breast [22,23], and oral cancers [24]. Grape seed extract (GSE), a rich source of proanthocyanidin, was found to be a potent inhibitor of aromatase activity, an enzyme expressed in higher levels in cancerous than in normal breast tissues [23]. GSE also induced apoptosis of MCF-7 and MDA-MB-235 breast cancer cells through modulation of both Bcl-2 and MEK/ERK signalling pathways [22]. GSE and other flavonoid extracts were also found to induce apoptosis in colon, leukemia, and oral cancer cell lines via caspase-3 activation [15,18,24], and were reported to inhibit ornithine decarboxylase (ODC), an enzyme involved in epithelial tumor cell proliferation [13,25,26].

To further evaluate the potential relationship between oral cancer inhibition and PAC, we sought to identify any specific or dose-dependent effects that may exist between the administration of GSE-derived PAC and the proliferation of oral cancers. In this way, we hope to more definitively characterize the effects of this non-toxic agent for its chemopreventive or chemotherapeutic value. Quantitative and dose-dependent relationships between PAC administration and oral cancer proliferation could, therefore, be established prior to the identification and elucidation of the pathways involved, and prior to animal and *in vivo *studies of this compound.

Based upon the observation that flavonoid-rich extracts, including PAC, inhibited proliferation of multiple tumor cell lines, this study examined the potential for PAC to modulate the proliferative phenotype of OSCC *in vitro*. While the aforementioned studies have increased our understanding of flavonoid- and nutrition-based inhibition of oral cancer proliferation, none to date have adequately investigated the role of PAC in modulating the proliferative phenotype of OSCC. This study tested the hypothesis that PAC modulates, in a dose-dependent manner, the proliferative phenotype of OSCC *in vitro*.

We determined that PAC significantly reduced the proliferation of the OSCC cell line, CAL 27, *in vitro*. Furthermore, this inhibition was dose-dependent and was more pronounced in this oral cancer cell line than in either of the cervical cancer cell lines that we evaluated, Ca Ski and GH354. In addition, the maximal growth inhibitory concentration (GI_MAX_) of PAC was sufficient to induce a net loss of cells (cytotoxic) in CAL 27, but not in either Ca Ski or GH354 cell lines.

Our previous work established that infection of OSCC with high-risk HPV 16 strongly promoted the proliferative potential of OSCC *in vitro *[27]. Therefore, we further sought to determine if PAC was capable of modulating HPV-driven OSCC proliferation *in vitro*. Our results provide one of the first demonstrations of HPV modulating the OSCC proliferative response to PAC. We also found that PAC inhibited HPV-positive OSCC proliferation and that the inhibitory concentration of PAC, providing the maximal proliferation inhibition and cytotoxicity (GI_MAX_), was the same for both HPV-positive and HPV-negative OSCC. These results suggest that PAC may exhibit selective inhibition of OSCC proliferation and that PAC administration may be sufficient to inhibit the increased proliferation of OSCC in the presence of HPV 16.

Standard chemotherapies and radiation treatment, frequently used to treat oral cancer patients, often have harsh and severe side effects that include tissue damage and malfunction. The discovery and validation of novel treatments and prevention strategies, lacking these harmful side effects, is one of our main short-term goals. This report provides evidence that PAC may provide one possible complementary or alternative therapy for oral cancer patients, as a safe, effective, cost-saving, and non-toxic treatment. We propose that this study provides compelling *in vitro *data to justify the exploration and elucidation of the mechanisms of PAC-induced proliferation inhibition of oral cancers and for testing of PAC in animal models.

## Methods

### Materials

Grape seed proanthocyanidin (PAC) extract (Lot #3717HF7361) was obtained from GNC Preventive Nutrition^® ^(Pittsburgh, PA). This commercial source of PAC was extracted from U.S. grown wine grapes, *Vitus vinifera*. Such commercial sources of grape seed PAC extract are demonstrated to contain 95% PAC and contain approximately 80–90% oligomeric PACs, including dimers, trimers, tetramers, and a small amount of monomeric flavonoids [28,29].

### Cell lines and cell culture

Human oral squamous cell carcinoma (OSCC) CAL 27, human cervical carcinoma (CC) Ca Ski, human cervical adenocarcinoma (CAC) GH354, and human foreskin fibroblasts (hFF) Hs27 cell lines were obtained from American Type Culture Collection (ATCC: Manassas, VA). CAL 27, GH354, and Hs27 cells were maintained in Dulbecco's Modified Eagle's Medium (DMEM) with 4.0 mM L-glutamine adjusted to contain 3.7 g/L sodium bicarbonate, 4.5 g/L glucose, and 110 mg/L sodium pyruvate, obtained from HyClone (Logan, UT). Ca Ski cells were maintained in RPMI-1640 medium with 2 mM L-glutamine, adjusted to contain 1.5 g/L sodium bicarbonate, 4.5 g/L glucose, 10 mM HEPES, and 1.0 mM sodium pyruvate, obtained from ATCC. Media was supplemented with 1% Penicillin (10,000 units/mL)-Streptomycin (10,000 μg/mL) solution and 10% fetal bovine serum (FBS), obtained from HyClone, except that GH354 was supplemented with 20% FBS. Cells were cultured in 75 cm^2 ^BD Falcon tissue-culture treated flasks (Bedford, MA) at 37°C and 5% CO_2 _in humidified chambers.

### Transfection

CAL 27 cells were seeded in 25 cm^2 ^BD Falcon tissue-culture treated flasks in appropriate media as described above and allowed to achieve 70% confluence. Cells were then transiently transfected by adding 1 μg/mL of the full-length HPV type 16 virus, cloned into the pBluescript SK-vector (ATCC #45113). The transfection was performed using the Stratagene Mammalian Transfection Kit (La Jolla, CA) according to the manufacturer's recommended protocol for CaPO_4 _transfection, and as previously described [27]. Transfected CAL 27 cells (CAL 27-TF16) were maintained in the same cell culture conditions as described above for non-transfected CAL 27 cells. Mock transfectants of CAL 27 (CAL 27-mTF) were also established by performing the transfection protocol, but without using virus.

### Microscopy of cell morphology and viability

Cells were visualized with a Zeiss Axiovert 40 inverted microscope (Gottingen, Germany), and images were captured at 100× magnification with a Canon PowerShot G6 digital camera (Tokyo, Japan). Digital images were subsequently processed using Adobe Photoshop (San Jose, CA) Image Analysis tools. In brief, several wells of unstained Ca Ski, GH354, CAL 27, CAL 27-TF16, and Hs27 cells were photographed at each time point of the proliferation assays (day 1 – day 4) and at each concentration of PAC (0 – 100 μg/mL) to visualize any effects on cell morphology. In addition, several wells of cells were also fixed at these time points using 50 μL of 10% buffered formalin and subsequently stained using crystal violet 1% aqueous solution (Fisher Scientific: Fair Lawn, NJ) to quantitatively document cell morphology, percent of cell spreading, and confluence. Additionally, at each time point, several wells were stained using Trypan Blue (Sigma: St. Louis, MO) and live cells were enumerated to determine viability, as previously described [30,31].

### Proliferation

*In vitro *proliferation assays of CAL 27, CAL 27-mTF, CAL 27-TF16, Ca Ski, GH354, and Hs27 cells were performed in the appropriate complete media, with and without the addition of PAC. The total concentrations of PAC used were between 10 and 100 μg/mL. Cells were plated in Corning Costar 96-well assay plates (Corning, NY) at a density of 1.2 × 10^4 ^cells per well, maintained at 37°C and 5% CO_2 _in humidified chambers, and their proliferation was measured over four days. Cultured cells were fixed every 24 hrs (day 1 – day 4) using 50 μL of 10% buffered formalin, and were stained using crystal violet 1% aqueous solution. The relative absorbance was measured at 630 nm using a Bio-Tek ELx808 microplate reader (Winooski, VT). Data were analyzed and graphed using Microsoft Excel (Redmond, WA). Three separate, independent replications of each experiment were performed.

### Statistics

The differences between treatments were measured using a *t *distribution, α = .05. All samples were analyzed using two-tailed *t *tests as departure from normality can make more of a difference in a one-tailed than in a two-tailed *t *test. As long as the sample size is even moderate (>20) for each group, quite severe departures from normality make little practical difference in the conclusions reached from these analyses [32].

### RT-PCR

RNA was isolated from 1.5 × 10^7 ^cells of CAL 27, CAL 27-mTF, and CAL 27-TF16, using ABgene Total RNA Isolation Reagent (Epsom, Surrey, UK) and the procedure recommended by the manufacture. RT-PCR was performed with the ABgene Reverse-iT One-Step RT-PCR Kit (ReadyMix Version) and a Mastercycler gradient thermocycler (Eppendorf: Hamburg, Germany) using the following primers synthesized by SeqWright (Houston, TX): HPV16 forward primer, ATGTTTCAGGACCCACAGGA; HPV16 reverse primer, CCTCACGTCGCAGTAACTGT. One μg of template RNA was used for each reaction. The reverse transcription step ran for 30 min at 47°C, followed by denaturation for 2 min at 94°C. Thirty-five amplification cycles were run, consisting of 20 sec denaturation at 94°C, 30 sec of annealing at 58°C, and 6.5 min of extension at 72°C. Final extension was run for 5 min at 72°C. Reaction products were separated by gel electrophoresis using Reliant 4% agarose gels (Cambrex: Rockland, ME). Bands were visualized by UV illumination of ethidium-bromide-stained gels and captured using a Kodak Gel Logic 100 Imaging System and 1D Image Analysis Software (Eastman Kodak: Rochester, NY).

### DNA isolation

DNA was isolated from 1.5 × 10^7 ^CAL 27 and CAL 27-TF16 cells, cultured with and without PAC, with the GenomicPrep DNA isolation kit (Amersham Biosciences: Buckinghamshire, UK), using the procedure recommended by the manufacturer. DNA was separated by gel electrophoresis using Cambrex Reliant 1% agarose gels and the bands were visualized by UV illumination of ethidium-bromide-stained gels and captured using a Kodak Gel Logic 100 Imaging System and 1D Image Analysis Software.

## Results

### Cell morphology

**Figure 1:**
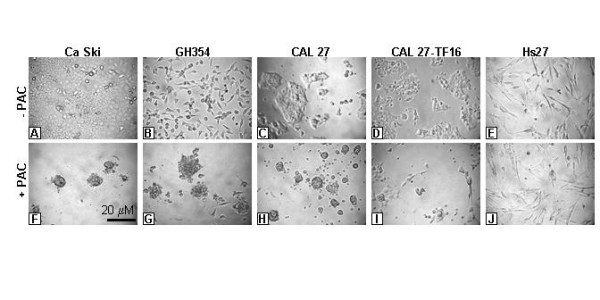
**PAC-induced effects on cell morphology *in vitro***. Cell lines were cultured in the absence (A-E) or presence (F-J) of PAC (50 μg/mL). Ca Ski (A,F) and GH354 (B,G) cervical cancers increased in absolute number and ratio of cell spreading without PAC (A,B) but displayed morphological features characteristic of apoptosis under PAC treatment (F,G). CAL 27 (C,H) and HPV 16-transfected CAL 27 cells (D,I) also grew in the absence of PAC (C,D), but demonstrated surface blebbing activity and morphological changes similar to cervical cells under PAC treatment (H, I). Hs27 normal fibroblast controls (E,J) grew in the absence of PAC (E) and less rapidly in the presence of PAC (J), without demonstrable microscopic evidence of apoptotic cellular features.

To examine any anti-cancer properties of grape seed-derived PAC on oral cancers, we began these experiments with a series of *in vitro *cell culture experiments designed to identify morphologic or culture-based effects of PAC, first on Ca Ski and GH354 cervical cancer cell lines, and subsequently on CAL 27 and HPV 16-transfected CAL 27 OSCC cell lines, as well as Hs27 normal human foreskin fibroblast cell line controls (Fig. 1). We utilized PAC working concentrations of 10, 50, and 100 μg/mL, similar to the concentrations used in other flavonoid- and GSE-based assays of oral cancer proliferation [17,24].

Our results demonstrated that both human cervical carcinoma lines, Ca Ski and GH354 (Fig. 1A, 1B), exhibited PAC-related changes in cell shape and cell structure at each concentration of PAC indicative of apoptotic changes, although these effects were most pronounced at 50 μg/mL (Fig. 1F, 1G). Cells rounded together and were characterized by substantial cell-surface blebbing, typical features of epithelial cancers undergoing morphological alterations associated with apoptosis [5]. Many of these cervical cancer cells further demonstrated the apparent rupturing of blisters, generating the appearance of a swollen, granular version of the original cells. In addition, there was a proportional reduction in the number of cells, the percent of spreading, and cell viability with the increased dose of PAC (Table 1).

**Table 1:** PAC-induced effects on cell viability, cell spreading, and confluence

	**- PAC [0 μg/ml]**	**+ PAC [50 μg/ml]**	**% change**
**Cell viability (%)**			
Ca Ski	98	10	-88
GH354	96	15	-81
CAL 27	81	10	-71
CAL 27-TF16	95	27	-68
Hs27	99	73	-26

**Cell spreading (%)**			
Ca Ski	99	5	-94
GH354	63	5	-58
CAL 27	85	5	-80
CAL 27-TF16	98	10	-88
Hs27	73	68	-5

**Confluence (%)**			
Ca Ski	88	23	-74
GH354	50	26	-48
CAL 27	45	15	-67
CAL 27-TF16	51	11	-78
Hs27	56	25	-55

An examination of the OSCC cell line, CAL 27, revealed PAC-related changes in morphology that paralleled the changes that were observed for both cervical cancer cell lines (Fig. 1C, 1H). The overwhelming majority of attached, spreading cells that were seen in the control were absent in the PAC experimental wells, with most PAC-treated cells exhibiting an apoptosis-like morphology, including rounded clustering of cells with surface blebbing and bubbling. In addition, many cells appeared to be detaching from the growth substrate or were found as floating cells which could be recovered from the culture medium. PAC also demonstrated reduction in the number, spreading, and viability of CAL 27 cells observed in each assay (Table 1).

Our previous work with CAL 27 demonstrated that transfection with HPV 16 increased the proliferation and induced the spreading of these cells, even when plated at low density [27]. In this study, CAL 27-TF16 cells (Fig. 1D) demonstrated a more heterogeneous mixture of cellular morphologies in the presence of PAC (Fig. 1I) than was observed for non-transfected CAL 27. Some CAL 27-TF16 cells retained typical spreading, adherent morphologies, while a smaller proportion of cells exhibited morphologies characteristic of the initial stages of apoptosis. These early stage-specific features included smaller groupings of rounding, clustering cells, cell-surface microspikes, and blebbing. A less pronounced reduction in cell spreading and cell viability was observed for CAL 27-TF16 (Table 1).

The non-cancerous fibroblast control cell line, Hs27 (Fig. 1E), demonstrated the ability to proliferate and spread in the presence of PAC at all concentrations (Fig. 1J). In addition, these cells did not exhibit characteristics of apoptosis; however, a reduction in the overall number of cells was evident at the higher concentration. This apparent inhibition of cellular proliferation was sustained and became more pronounced over time, although these effects on cell viability and cell spreading were less pronounced than for any of the other cell lines tested (Table 1).

### Proliferation

#### Ca Ski (CC) and GH354 (CAC)

To quantitate our observation that the administration of PAC may inhibit the proliferation of these cancer cell lines, in addition to the possible induction of apoptosis, the cervical carcinoma cell lines, Ca Ski and GH354, were grown in 96-well assay plates and their proliferation was measured over four days in three separate, independent experiments. Our results demonstrated that PAC elicited selective responses in the Ca Ski (HPV 16-containing) and GH354 (HPV 18-containing) cell lines. Low dose administration of PAC stimulated Ca Ski proliferation, while higher doses were inhibitory in a dose-dependent manner. All doses of PAC were inhibitory in GH354 cells, although the level of inhibition was determined to be dose dependent.

**Figure 2:**
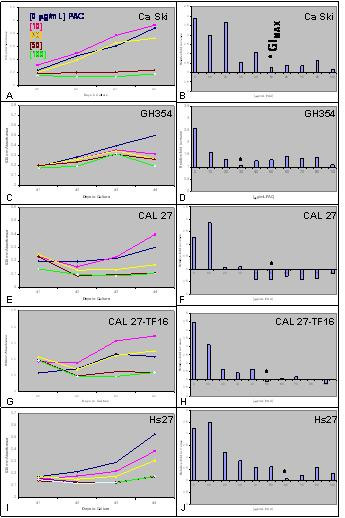
**PAC-induced effects on cell proliferation *in vitro***. 96-well assay plates with media containing 10% fetal bovine serum (FBS) in the absence and presence of increasing PAC dosages [0–100 μg/mL] and cells were allowed to proliferate for four days. The addition of PAC at low concentration (10 μg/mL) stimulated proliferation of Ca Ski (A), CAL 27 (E) and CAL 27-TF16 (G) cells, but inhibited proliferation of GH354 (C) and Hs27 (I) cells. Measurement of the relative change in proliferation between day 4 and day 1 revealed that the maximal PAC growth inhibition dose (GI_MAX_) was between 30–60 g/mL for all cell lines tested (B,D,F,H,J).

More specifically, Ca Ski cell proliferation was initially stimulated by the administration of PAC at the concentration of 10 μg/mL (+5%), while higher doses, between 20–100 μg/mL, had an opposite, inhibitory effect on proliferation (Fig. 2A). To reduce the initial proliferation-stimulating effects of trypsinizing and plating the cells into each assay, seen between day 0 and day 1 in most assays, we determined the relative change in proliferation between day 4 and day 1 (day 4 minus day 1). Our results demonstrated that the effects of PAC induced only a slight inhibition of cell proliferation between 10–20 μg/mL, but induced a sharp decrease in proliferation extending over the range of 30–100 μg/mL with the maximal growth inhibitory dose (GI_MAX_) at 50 mg/mL (Fig. 2B).

The effects of PAC administration on GH354 cells produced slightly different proliferative responses compared with the effects on Ca Ski cell proliferation (Fig. 2C). For example, all doses of PAC tested inhibited GH354 proliferation. Although these results suggest that the lowest dose of PAC (10 μg/mL) had the smallest effect of inhibiting proliferation, an analysis of the relative change in proliferation revealed a more complicated interaction between PAC effects and GH354 proliferation (Fig. 2D). These results revealed that all doses of PAC tested were growth inhibitory; GI_MAX _was established at 30 μg/mL, with higher doses exhibiting slightly less robust proliferation-inhibitory effects.

#### Hs27 (hFF)

A control cell line, Hs27, human foreskin fibroblasts, was also tested as described above. Our results demonstrated that Hs27 cells exhibited dose-dependent responses to PAC administration (Fig. 2I). GI_MAX _was determined to be 60 μg/mL, which closely approximated the maximum inhibitory dosages observed for the other cell lines tested (Fig. 2J). As with Ca Ski and GH354 cell lines, no dosage of PAC resulted in a net loss of cells over the course of these assays.

#### CAL 27 (OSCC) and CAL 27-TF16 (OSCC)

To test our hypothesis that PAC administration inhibits proliferation of OSCC, the OSCC cell line, CAL 27, was assayed and proliferation was measured over four days in three, separate independent experiments. Our results demonstrated that PAC induced variable responses, similar to the effects on Ca Ski cells, in a dose-dependent manner (Fig. 2E). The proliferation of CAL 27 cells was slightly increased by the lowest concentration of PAC (10 μg/mL), while increasing doses produced an inhibitory effect on proliferation over the range of 20–100 μg/mL (Fig. 2F). To determine the growth inhibitory dose, we analyzed the relative change in proliferation and found that PAC doses between 20–40 μg/mL exhibited increasing inhibitory effects on proliferation, GI_MAX _was determined to be 50 μg/mL, while larger doses, between 60–80 μg/mL, displayed ever-diminishing inhibitory effects as the dose was increased. All PAC doses above 40 μg/mL resulted in a net loss of cells over the course of the assay.

Our previous studies with OSCC proliferation revealed that CAL 27 cells proliferated more rapidly after transfection with HPV 16 [27]. To contextualize our results from this current study, we assayed HPV 16-transfected CAL 27 cell proliferation under PAC administration over four days in three, separate independent experiments. Our results revealed that PAC induced dose-dependent effects, similar to the responses of CAL 27, with some notable differences (Fig. 2G). The proliferation of CAL 27-TF16 cells was increased by the two lowest concentrations of PAC (10–20 μg/mL), while further increasing doses produced inhibitory effects on proliferation within the range of 30–100 μg/mL. GI_MAX _was also determined to be 50 μg/mL, with higher doses exhibiting no greater inhibitory effects than at GI_MAX _(Fig. 2H). The maximal inhibitory PAC dose of 50 μg/mL resulted in a net loss of these cells over the course of the assay.

**Figure 3:**
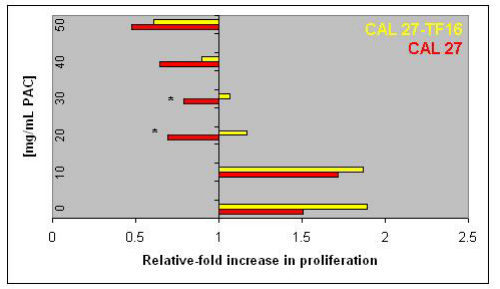
**HPV 16 mediates the proliferation-inhibitory effects of PAC on OSCC**. PAC dosage of 50 μg/mL was the GI_MAX _for both CAL 27 and CAL 27-TF16 cells. PAC doses greater than 20 μg/mL resulted in a net loss of cells in the CAL 27 assays, but was modulated by the presence of HPV 16 at dosages above 40 μg/mL in the CAL 27-TF16 assays.

To clearly delineate the results of our proliferation assays for CAL 27 and CAL 27-TF16, cells were plated in the presence of PAC dosages between 0 and GI_MAX _(50 μg/mL). Confirming our previous observations, PAC administration once again stimulated CAL 27 cells at the lowest concentration tested (10 μg/mL), but higher concentrations inhibited proliferation (Fig. 3). All concentrations above 20 μg/mL resulted in a net loss of cells, with the GI_MAX _observed at 50 μg/mL, demonstrating a net loss of more than 50% of the original number of cells plated at day 0. PAC administration also stimulated CAL 27-TF16 cells at low concentrations (10 μg/mL), but higher concentrations induced reduced levels of proliferation. One disparate result was that PAC administration between 20–30 μg/mL inhibited CAL 27-TF16 cells, unlike the non-transfected CAL 27 cells, without precipitating a net loss of cells. However, PAC dosages above 40 μg/mL did result in a net loss of cells with GI_MAX _determined in this assay to be 50 μg/mL.

### Dose-dependent cell morphology responses

**Figure 4:**
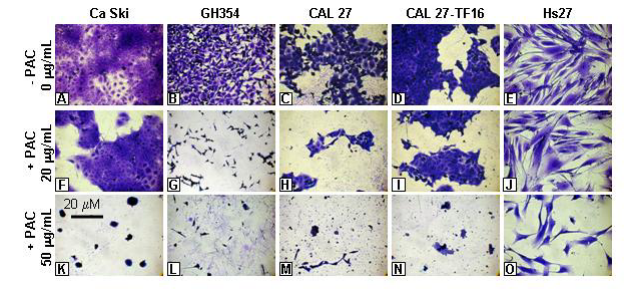
**Dose-dependent effects of PAC on proliferation and morphology**. Cells were plated in 96-well assay plates in media with FBS. The upper row (A-E) was grown without PAC, the mid-level row (F-J) in the presence of PAC at 20 μg/mL, and the lower row (K-O) under PAC administration at 50 μg/mL. All cell lines exhibited a dose-dependent response to PAC administration, with higher doses resulting in fewer cells at day 4. Ca Ski (A), CAL 27 (C), CAL 27-TF16 (D) and Hs27 (E) cells were reduced in number with PAC at 20 μg/mL (F, H, I, J), unlike GH354 cells (B), which experienced a reduction in proliferation, and morphological changes indicative of apoptosis (G). Cellular proliferation was reduced by PAC in all cell lines (K-O) at 50 μg/mL; all cell lines exhibited altered morphology, except Hs27 (O) cells which appeared normal, although fewer in number.

Even though we have described the dose-dependent inhibition of cellular proliferation in response to PAC administration through *in vitro *cell culture assays, we wished to clarify the effects of PAC administration on cell morphology at dosages that may provide the most information about the nature and timing of possible apoptosis events (Fig. 4). We first examined cell growth in all cell lines in the absence of PAC for comparison (Fig. 4A–E).

Inhibition of proliferation was induced by treatment of cells with low concentrations of PAC, sustained over time (Fig. 4F–J). In examining the cervical carcinoma cell lines, Ca Ski and GH354, we observed that cell number was reduced in Ca Ski cultures (Fig. 4F), but that both morphology and cell number were altered in GH354 cells (Fig. 4G) at low PAC concentration (20 μg/mL). The same concentration of PAC reduced the overall number of Hs27 cells, without inducing demonstrable changes in cell morphology (Fig. 4J). The OSCC cell lines, CAL 27 (Fig. 4H) and CAL 27-TF16 (Fig. 4I), also exhibited reduced proliferation in response to this PAC concentration, but exhibited no specific evidence of apoptotic events.

Higher concentrations of PAC, on the other hand, elicited different responses in cell morphology and appearance. Cell clustering and rounding was induced in all cancer cell lines (K-N) with PAC (50 μg/mL). Possible evidence of apoptosis at this concentration was first observed in all cell lines, except Hs27 (Fig. 4O), between 18 and 24 hours, and was observed as a relatively slow event that resulted in a rounding of cells together followed by intense surface blebbing. Surface blebbing continued from day 1 to day 4 until more active blistering and lytic events resulted in the net loss of cells in each assay.

The cervical carcinoma cell line, Ca Ski (Fig. 4F), and HPV16-transfected OSCC cell line, CAL 27-TF16 (Fig. 4I), were more resistant to lysis and cell death at the lower PAC concentration (20 μg/mL) and appeared to be rounded and blebbing at day 4, while the majority of GH354 cells (Fig. 4G) and non-transfected CAL 27 cells (Fig. 4H) may have already undergone these transformations. The higher PAC concentration (50 μg/mL approximating GI_MAX_) further reduced absolute cell number and induced these morphologic changes in all cell lines (Fig. 4K–N), except Hs27 cells which exhibited no evidence suggesting apoptotic changes (Fig. 4O).

### Nucleic acid analysis

**Figure 5:**
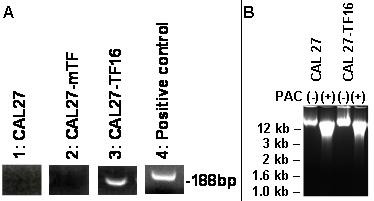
**Total RNA and DNA analysis of CAL 27 cells**. RT-PCR performed on total RNA extracted from OSCC, CAL 27, revealed that CAL 27 did not express HPV 16-specific mRNA (A: Lane 1). Mock transfectants (A: Lane 2) also did not express HPV. HPV 16-transfected cells (A: Lane 3) expressed HPV 16; specific primers were tested using PCR and the full-length HPV 16-DNA template (A: Lane 4). Total DNA isolated from CAL 27 cells (B: Lane 1) and CAL 27-TF16 cells (B: Lane 3) demonstrated DNA alterations following PAC treatment (50 μg/mL), including a more diffuse banding pattern and lower molecular weight, possibly indicating the onset of DNA fragmentation among the treated cells (B: Lane 2, 4).

To confirm that the CAL 27 cells used in this study did not harbor HPV 16 prior to transfection, we performed RT-PCR on total RNA isolated from cultured CAL 27 cells used for these experiments and oligonucleotide primers specific for the HPV 16 strain (Fig. 5A). Our results demonstrated that neither CAL 27 cells (Lane 1) nor CAL 27 mock-transfected cells (Lane 2) expressed HPV 16 mRNA prior to transfection. After completing transient transfection of CAL 27 with HPV 16, our results confirmed that CAL 27-TF16 cells expressed HPV 16-specific mRNA (Lane 3). HPV 16-specific primers were tested using PCR and the HPV 16 DNA template (Lane 4) to confirm the band size and specificity of the primers to the template.

The administration of PAC had multiple effects, including a dose-dependent reduction in cellular proliferation and changes in cellular morphology among cancer cell lines. This study also assessed total DNA isolated from CAL 27 and CAL 27-TF16 cells in the absence and presence of PAC at 50 μg/mL (Fig. 5B), revealing that PAC administration induced some alteration and fragmentation of total DNA, resulting in a more diffuse and less compact banding pattern. In addition, PAC administration reduced the overall molecular weight of the chromosomal DNA extracted, which may represent one stage in the process of DNA fragmentation, characteristic of cells undergoing necrosis or apoptosis.

## Discussion

Proanthocyanidins are part of a larger group of compounds that have been demonstrated to have anti-proliferative and anti-tumor effects [13,26,33]. PAC can be isolated from multiple sources and the therapeutic benefits of PAC may be determined, in part, by the fruit or vegetable from which it is derived [34]. GSE and other fruit-derived flavonoid extracts have demonstrated robust anti-proliferative effects on other oral and epithelial cancers [17,24,35,36].

Although other studies have demonstrated that particular fractions of GSE or raspberry extracts may be cytotoxic against oral tumor cell lines, none have yet examined PAC specifically to analyze its effects on oral cancer proliferation. Through this study, we have demonstrated that PAC exhibited dose-dependent inhibitory and cytotoxic effects on cervical cancer cell lines, with these effects more selective and intensely specific for OSCC. In addition, HPV, now believed to initiate oral cancers among non-smokers, and to modulate OSCC proliferation and progression among smokers, was modulated and reduced, in a dose-dependent manner, by the administration of PAC.

A closer examination of PAC administration among these various cell lines revealed that the effects of PAC were less intense and more gradual in the foreskin and cervical cancer cell lines, Hs27 and Ca Ski, respectively. In contrast, at even the lowest dosages, PAC substantially inhibited the proliferation of both the cervical cancer cell line, GH354, and the OSCC cell line, CAL 27. Furthermore, the level of inhibition was dissimilar between these two cancers – only limiting proliferation of the cervical cell line, GH354, but resulting in a net loss of cells in the oral cancer line, CAL 27. This level of cytotoxicity may represent a selective effect which suggests that OSCC may be rendered more susceptible to this treatment.

Although we found that PAC exhibited a general inhibitory effect on all cell lines tested, a morphological change was observed after PAC administration in both the cervical and oral cancer cell lines that suggested the possible induction of apoptosis. Images obtained from live cell cultures under PAC administration demonstrated that many of the cells were clustering, rounding, and blebbing – morphologic changes that are often suggestive of apoptosis. However, once the cells were fixed and stained to quantify these effects on proliferation, only Ca Ski cells and non-cancerous controls Hs27 were sufficiently adhered to remain in the assay plates at the GI_MAX _concentration of PAC. One possible reason for this phenomenon may be the degree of progression toward apoptosis or cell death; the more susceptible cell lines, such as GH354, CAL 27, and CAL 27-TF16, could be further along the apoptotic pathway and therefore less capable of adhering and more likely to be removed by mechanical forces during the fixation and staining process. The DNA analysis revealed fragmentation, but no banding patterns indicative of internucleosomal cleavage, suggesting further experimentation will be necessary to delineate the pathways and mechanisms associated with these changes.

Aside from demonstrating that PAC may exhibit differential effects among different cancers, we found convincing evidence that HPV may modulate, and in turn be modulated by, the effects of PAC with respect to the OSCC proliferative response. Our previous research found that HPV 16 strongly induced OSCC proliferation and our findings here indicate that HPV 16 also influenced OSCC response under PAC administration. The introduction of HPV 16 into these cells not only increased their relative levels of proliferation, but also mediated their response to PAC, with lower dosages actually stimulating proliferation of CAL 27-TF16 cells. Interestingly, the GI_MAX _was determined to be invariable for both transfected and non-transfected cells, suggesting that despite this modulating effect, the HPV 16-specific effect was not sufficient to alter OSCC proliferation in response to incremental increases of PAC administration at or beyond GI_MAX_.

One critical goal of cancer treatment is to find chemopreventive and therapeutic agents that selectively target cancer cells without detrimental effects to healthy cells and tissues. Although many chemotherapeutic agents are currently being used in the treatment of oral cancers, such as cisplatin (CDDP) and 5-fluorouracil (5-FU), it is known that most tumors may be more or less resistant to these drug therapies because they are not dependent on a single receptor or signal transduction pathway for growth and progression [5]. Thus, compounds or combinations of compounds that may activate multiple apoptotic pathways, such as PACs, may provide new and more effective treatment regimens for patients with oral cancer.

Recent studies have determined that PACs can be administered orally as a dietary supplement and are subsequently bioavailable, in both serum and tissues at μg/mL concentrations, without any significant toxicity, implying that PACs may represent one class of promising candidates for use as an adjuvant therapeutic agent in patients receiving chemotherapy for oral cancer [37,38]. The supporting evidence that GSE and other flavonoid extracts are effective as adjuvants and treatments for breast, colon, and prostate cancer, combined with the results of this study, demonstrate that PAC may act even more selectively on OSCC than other cell lines. This suggests the possibility that PACs may act synergistically to induce selective inhibition and apoptosis of oral cancers *in vivo*.

## Conclusion

PAC-containing commercial products, such as ActiVin^®^, OptiBerry^®^, Grape Seed Proanthocyanidin Extract, and others, have been verified as well-tolerated and non-toxic [33,34,39]. However, due to incomplete information regarding alternative and combination therapies, we advocate here for systematic animal, and subsequently human chemoprevention trials, to confirm that PAC may suppress tumor proliferation or induce tumor apoptosis, without eliciting deleterious effects on viability and growth of normal tissue. This study provides convincing evidence that PAC administration is sufficient to reduce OSCC proliferation *in vitro *and that it may also effectively modulate the proliferation-enhancing effects of concomitant HPV infection. Consequently, if these effects can be substantiated, it will then become vital to decipher which of the signalling pathways PAC may influence in OSCC, including the possible inhibition of MAPK (mitogen activated protein kinase), ODC (ornithine decarboxylase), EGFR (epidermal growth factor receptor), or caspase-dependent pathways.

## Competing interests

The author(s) declare that they have no competing interests.

## Authors' contributions

KK and SO conceived, monitored and coordinated the experimental design. SO, MK, KC, DF, DJ, JP and AS carried out the transfection, cell culture, and proliferation assays. Both KK and SO contributed equally to the writing of this manuscript. All authors have read and approved the final manuscript.

## Pre-publication history

The pre-publication history for this paper can be accessed here:


